# Informed consent in paediatric critical care research – a South African perspective

**DOI:** 10.1186/s12910-015-0052-6

**Published:** 2015-09-09

**Authors:** Brenda M. Morrow, Andrew C. Argent, Sharon Kling

**Affiliations:** 1 Centre for Medical Ethics and Law, Department of Medicine, Faculty of Medicine and Health Sciences, Stellenbosch University, PO Box 241, Cape Town, 8000 South Africa; 2 Department of Paediatrics and Child Health, University of Cape Town, 5th Floor ICH Building, Red Cross War Memorial Children’s Hospital, Klipfontein Rd, Rondebosch, Cape Town 7700 South Africa; 3 Paediatric Intensive Care Unit, Red Cross War Memorial Children’s Hospital, Klipfontein Rd, Rondebosch, Cape Town 7700 South Africa; 4 Department of Paediatrics and Child Health, Faculty of Medicine and Health Sciences, Stellenbosch University, Box 241, Cape Town, 8000 South Africa

## Abstract

**Background:**

Medical care of critically ill and injured infants and children globally should be based on best research evidence to ensure safe, efficacious treatment. In South Africa and other low and middle-income countries, research is needed to optimise care and ensure rational, equitable allocation of scare paediatric critical care resources.

Ethical oversight is essential for safe, appropriate research conduct. Informed consent by the parent or legal guardian is usually required for child research participation, but obtaining consent may be challenging in paediatric critical care research. Local regulations may also impede important research if overly restrictive.

By narratively synthesising and contextualising the results of a comprehensive literature review, this paper describes ethical principles and regulations; potential barriers to obtaining prospective informed consent; and consent options in the context of paediatric critical care research in South Africa.

**Discussion:**

Voluntary prospective informed consent from a parent or legal guardian is a statutory requirement for child research participation in South Africa. However, parents of critically ill or injured children might be incapable of or unwilling to provide the level of consent required to uphold the ethical principle of autonomy. In emergency care research it may not be practical to obtain consent when urgent action is required. Therapeutic misconceptions and sociocultural and language issues are also barriers to obtaining valid consent.

Alternative consent options for paediatric critical care research include a waiver or deferred consent for minimal risk and/or emergency research, whilst prospective informed consent is appropriate for randomised trials of novel therapies or devices.

**Summary:**

We propose that parents or legal guardians of critically ill or injured children should only be approached to consent for their child’s participation in clinical research when it is ethically justifiable and in the best interests of both child participant and parent. Where appropriate, alternatives to prospective informed consent should be considered to ensure that important paediatric critical care research can be undertaken in South Africa, whilst being cognisant of research risk. This document could provide a basis for debate on consent options in paediatric critical care research and contribute to efforts to advocate for South African law reform.

## Background

It has been observed that the need for intensive care is increasing globally, with a disproportionate burden of critical illness in low and middle-income countries, where access to intensive care is particularly limited [1]. A 2007 audit of intensive care services in South Africa highlighted this injustice. The available public sector intensive care unit (ICU) bed:population ratios varied from about 1:20 000 to 1:80 000 in different provinces [2] and there was a further inequity between paediatric and adult ICU facilities, with only 19.6 % of the nearly 4200 ICU beds in South Africa dedicated to paediatric and neonatal patients [2].

It is accepted that the medical care of critically ill and injured infants and children should be based on the best available research evidence in order to optimise patient outcome [3–9] and ensure rational and equitable use of scarce resources [10]. However clinical research in this population has been severely limited, particularly in low and middle-income countries including South Africa, due to both lack of prioritisation and concerns about protecting vulnerable children from potential harm in clinical trials [11–14]. This has led to the situation where some of the sickest, most vulnerable children are subjected to the most anecdotally based medical practice, including the use of untested, off-label or unlicensed medications [5, 11, 15–17].

It is not appropriate to extrapolate the results from animal and adult studies to determine appropriate paediatric treatment, owing to pathophysiological, anatomical and developmental differences amongst these groups [12, 18]. The risks of harm and the need to protect children have to be balanced against children’s consitutional rights to the safest, most efficacious available treatments, but this can only be determined by appropriate research [19–21], which requires reasonable access to the research population [15].

In order for research involving children to be approved, the child’s parent/s or authorised legal representative are required to provide informed consent for their child’s participation [11, 12, 22]. For a number of reasons which will be explored in this article, obtaining comprehensive, voluntary prospective informed consent may be particularly challenging in both emergency situations and paediatric research [6]. When these circumstances combine, as in paediatric critical care research; obtaining consent may be even more difficult [6].

By synthesising and contextualising the results of a comprehensive literature review, this paper describes the underlying ethical principles and regulatory requirements for paediatric critical care research in South Africa, the potential barriers to obtaining written informed consent in the context of a critically ill or injured child, and the options for obtaining or not obtaining consent in this context. It is hoped that this document could provide a basis to stimulate debate around consent options in paediatric critical care research and contribute to efforts to advocate for South African law reform.

For the purposes of this paper, paediatric participants refer to children from beyond the neonatal period to age 17; research risk refers to the potential that participants will experience harm from participating in clinical paediatric critical care research; harm refers to physical, psychological, spiritual or social injuries, discomforts or inconveniences [23]; and paediatric critical care refers to the health care of children with life- threatening medical conditions and injuries, and after major surgery [24].

## Discussion

### Ethical principles and research risk

The *Belmont Report* describes three fundamental ethical principles underpinning human research: respect for persons, beneficence and its corollary non- maleficence, and justice or fairness [3, 21]. Respect for persons in turn incoporates the principle that individuals should be treated as autonomous agents, and that persons with diminished autonomy are entitled to protection [21]. Young children may lack decision-making capacity, and are generally considered a relatively vulnerable population, owing to their developmental and cognitive levels and dependence on adults for care and protection [6, 12]. However, if one categorically protects this vulnerable group from any risk of harm, all paediatric research is effectively precluded. This is itself unethical as countless future children would be denied potential research benefits, including that of appropriate, evidence-based medical care [5].

An example of the effect of over-protection from research is evident in the current practice of administering drugs, untested in the paediatric population, to critically ill infants and children, thereby exposing patients to potentially harmful medications as standard practice. A consequentialist standpoint might allow almost any research where the potential benefits for future patients (social value) outweighs the risks to individual participants, regardless of risk level. This may, however, not be ethically justifiable, as it potentially exposes the most vulnerable patients to high risk of harm with no potential for individual benefit [23].

Ethical research can be conducted with vulnerable populations, including children, provided additional safeguards are in place to protect them from potential exploitation and harm [9, 20, 21, 25]. These safeguards include the “necessity” and “participant-condition” requirements, whereby the research cannot reasonably be performed in a non-vulnerable surrogate population and the participant must have the specific condition being investigated. Further safeguards, where possible, include independent participation monitors, to monitor individual children’s involvement, including changing risk profiles and responses (physical and emotional), throughout the research study; and independent consent monitors to assess the quality of informed consent [25].

Both researchers and research ethics committees (RECs) are obliged to protect child participants by minimising associated research risks [5]. Accordingly, different categories of research involving children have been described according to risk levels [3, 12, 23] (Table 1). It has been suggested that allowable research risk should be positively correlated with the participants’ ability to consent, and inversely proportional to vulnerability [26]. Therefore, the maximum research risk level to which critically ill or injured children may be exposed is necessarily lower than what would be allowed in adult participants [5]. In the United States, this threshold is set at “minor increase over minimal risk” (Table 1) [5].

**Table 1:** Categories of research risk [3, 12, 23]

Research category	Benefit	Example
1. Not greater than minimal risk interventions	● Unlikely to be individual benefit	Additional testing on routinely collected specimens
	● Likely to yield generalisable knowledge	
2. Interventions posing minor increase over minimal risk	● No direct benefit to individual child participant	Drawing blood for analysis
	● Likely to yield generalisable knowledge	
3. Interventions posing greater than minimal risk	● Possibility of direct benefit to individual child participant	Clinical drug trials
	● Will yield generalisable knowledge	

The United States Common Rule presents minimal risk as when “the probability and magnitude of harm or discomfort anticipated in the research is not greater in and of themselves than those ordinarily encountered in daily life or during the performance of routine… tests” [20]. There are a number of problems with this definition, including different standards for what constitutes “daily life” - a child in a violent, gang-ridden community in South Africa, for example, will be exposed to greater daily risks than a child living in a higher socioeconomic environment with less crime. A detailed discussion of these issues is beyond the scope of this paper. Nevertheless, if we use the definition above, virtually no studies comparing drugs, devices, or other critical care interventions would ever qualify as minimal risk [27].

We agree with previous authors that instead of evaluating the absolute risks of research, the incremental risks of research interventions should be judged above the risks the patient would experience as part of routine clinical care, as the daily life standard equivalent [7, 27, 28]. This approach would allow important paediatric critical care research to be conducted without unnecessary restrictions and bureaucracy [27] and is supported by the Common Rule statement that independent review boards should only evaluate those risks and benefits that may result from the research itself [20]. This requires careful separation of the risks of study interventions and those inherent to the condition being managed [5, 7].

Similar to clinical decision making processes, any research interventions for which the incremental benefits equal or exceed the risks are considered ethically acceptable [5, 29]. In some cases a small net risk may be allowable in order to generate knowledge to benefit future patients [5, 25].

In order to fully assess risk and benefit, researchers should distinguish between therapeutic and non-therapeutic research procedures [30, 31]. Therapeutic procedures, such as specific ventilation strategies or administration of medications, have the potential to benefit the study participant, but may also introduce harm. Non-therapeutic procedures, such as downloading data from monitors, are performed solely to answer the research question and the participant does not stand to directly benefit from these procedures, although the generated knowledge could inform clinical practice as well as future clinical trials, thus potentially benefiting future patients [3, 7].

In studies of therapeutic procedures the standard of clinical equipoise must be met. This requires that the research intervention be consistent with competent clinical care and that a genuine state of uncertainty exists amongst the expert medical community as to the preferred treatment [3, 7]. Sometimes the requirement for equipoise might hinder important research to confirm the efficacy of current (but unproven) treatments. For example, the Fluid Expansion as Supportive Therapy (FEAST) Trial, in which over 3000 critically ill children were randomised to different fluid resuscitation strategies [32], was challenged with regard to the presence of initial equipoise. Most clinicians from developed countries would present fluid boluses as the treatment of choice for shock in children, although this was not local standard practice, and the trial authors argued that there was insufficient evidence for fluid resuscitation of children with shock or life-threatening infection in resource-poor settings [33]. The trial was eventually able to report the surprising finding of a significant increase in the risk of dying in those who had received the fluid boluses compared to controls [32, 33]. These important, if controversial, findings would never have been identified if the trial had not been conducted due to apparent lack of equipoise.

Non-therapeutic interventions in paediatric critical care research can only be justified if they fall into one of the first two categories outlined in Table 1 [3]. However, it is sometimes difficult to distinguish between therapeutic and non-therapeutic research, e.g. therapeutic research may have many associated non-therapeutic interventions, such as blood draws for research purposes. The term “therapeutic” in itself may be misleading in the presence of clinical equipoise where a trial of a new therapy or medication offers only the potential for therapeutic benefit, at best. It is therefore suggested that, when evaluating research protocols, RECs consider the associated risk categories of the research as more important than the distinction between therapeutic and non-therapeutic research [34].

### Legal and regulatory issues in South Africa

Under South African law, children under 18 years of age are considered minors [35] and therefore unable to act independently without assistance from parents or legal guardians [36]. However, there are many legal discrepancies on when children evolve the capacity to make decisions for themselves. The amended Children’s Act No 38 of 2005 has attempted to resolve some of these [35]: children judged to have sufficient maturity and mental capacity can now consent to, amongst others, medical therapeutic procedures; human immunodeficiency virus (HIV) testing; and contraceptive use from the age of 12 years [35]. There are no clear legal statutes specifying when children can independently consent to participation in human research studies, but ethical norms require parental consent and, where possible, child assent for all children under 18 years of age [36].

In 2012, Section 71 of the National Health Act 61 of 2003 came into effect in South Africa, introducing new requirements for health research involving minors, including the requirement for written consent from parents or guardians for all research; that “therapeutic research” must be in the best interests of the participant; and that ministerial consent must also be obtained for all “non- therapeutic research” [37]. The revised Act was promulgated without accompanying guidelines or regulations for how ministerial consent was to be obtained and the new legal requirements were in conflict with the 2004 National Department of Health Research Ethics Guidelines [38]. Many South African RECs therefore continued procedures in accordance with the 2004 Department of Health Research Ethics Guidelines [38]. These guidelines state that research on children should be of minimal risk only, that consent from the parent or legal guardian is required in all but exceptional circumstances (such as emergencies), and consent is also required from the minor where possible [39]. In October 2014 ministerial consent was officially delegated to RECs registered with the National Health Research Ethics Council [40]. Interestingly, the South African Good Clinical Practice (GCP) Guidelines and the national Human Sciences Research Council (HSRC) guidelines allow a child’s “caretaker” to provide consent for their participation in research [41, 42]. This acknowledges the many South African children who are raised and cared for by people other than biological parent/s or legal guardians, often by an informal arrangement; however this provision is in direct conflict with the National Health Act 61 of 2003 [34]. Clearly there is an urgent need for alignment between the National Health Act and other South African research guidelines in order to ensure both legally and ethically acceptable practice by RECs and researchers.

### Barriers to obtaining informed consent for paediatric critical care research

Throughout the world, it is generally accepted that obtaining informed consent from a parent or legal guardian for their child to participate in paediatric research is necessary in order to protect the child from potential research-related harm and to protect parental autonomy [43].

Valid informed consent consists of three major elements: 1) adequate disclosure of information, which requires sufficient time; 2) adequate participant or proxy understanding of information received; and 3) voluntariness of the decision; without real or perceived threat or coercion, either explicit or implicit [44–47]. All three of these requirements may be threatened in the paediatric critical care context as it may be impractical to obtain informed consent; the validity of consent may be questionable; sociocultural barriers may impact on the ability to obtain consent; and the potential for therapeutic misconception is particularly high. Obtaining informed consent may also at times do more harm than good. These issues are discussed below, and the relevance to the South African context is highlighted.

#### Impracticability

In paediatric critical illness research, a parent or legal guardian’s absence or the need for urgent action may not provide the time needed for informed consent to be obtained prior to the research intervention, for example in resuscitation research [6, 13, 18, 26]. In such cases it might in fact be harmful to delay the research intervention in order to obtain consent [11]. In the context of emergency research in child victims of trauma (e.g. fires, motor vehicle accidents), which is very common in South Africa and other low and middle-income countries [48], the parent may have also been injured, or even died, in the incident, substantially impacting on the likelihood of informed consent and attaining recruitment goals.

A rural Ghanian genomics study (“MalariaGEN”) highlighted the complexity of seeking consent in emergency research situations. Children admitted to hospital with severe malaria, needing urgent and rapid medical treatment, were recruited as cases into the study. However, after recruiting initial cases, researchers felt that it was “practically impossible and ethically inappropriate to conduct a detailed consent process for research before collecting the samples needed for diagnosis and treatment” [49].

#### Validity of consent – situational incapacity

“…*you know being only one head my heart was shaken about how the child was… that’s why [my head] didn’t grab many things”.* Quote from a mother of a child enrolled in the Fluid Expansion as Supportive Therapy (FEAST) study [50].

Paediatric critical care services provide care to children with life-threatening medical and surgical conditions, and may involve care in a complex environment with state-of-the-art equipment. Critically ill children and their immediate families may be far removed from their traditional support structures for political, financial, or other reasons. This is particularly true of South Africa’s large migrant community.

Even in the best, most well resourced environments, having a child with severe illness or injury is extremely (dis) stressful for the child, parents and extended family [24, 51–53]. The illness often evolves rapidly, leaving little time for parents to assimilate the implications of their child’s disease before they may be approached to provide consent for both clinical procedures and research [3]. A number of studies suggest that the paediatric intensive care unit (PICU) environment is more stressful than that of a general paediatric hospital ward, and that the needs of parents and their critically ill infants and children are unique and different from those in neonatal or adult intensive care units [54–59]. Stress is defined here as “circumstances which place physical or psychological demands on the individual and the overall emotional reactions experienced by the individual or family” [60].

Parents of critically ill or injured children may be unable or unwilling to prioritise information about a research study over concern for their child’s immediate welfare. Studies have shown that even competent adults may not completely understand the research for which they have given consent [61–63]. The emotional and psychological burden on parents of critically ill or injured children may impair their capacity for rational informed decision making, including decisions relating to research [6, 11, 13, 53]. Parental anxiety (being “too stressed” or “overwhelmed”) has been reported to be the most common reason for declining participation in clinical PICU research [54, 64]. Some identified stressors include changes in the child’s appearance; life support equipment and monitors (with associated alarms); nursing procedures; fatigue; poor nutrition; lack of diurnal rhythms; and communication barriers between parents and healthcare staff [24, 65, 66].

A positive correlation between parental anxiety and the number of invasive procedures has been reported, which relates to acuity of illness [24, 51]. It has also been shown that parents of children admitted as emergencies have higher levels of stress than of those admitted electively [67], with higher consent rates in parents of children undergoing planned surgery than emergency admissions [64]. This is important in the context of a developing country such as South Africa, where the majority of PICU admissions are emergencies, mainly for the management of severe infectious diseases [10].

The “FEAST” trial incorporated a qualitative sub-study to investigate the consent process [50, 68]. This sub-study suggested that being asked to make choices about research at the time of admission of a critically ill child might cause harm by “raising concerns and doubts at a time when parents are unable to listen, ask, understand, or challenge those that they are seeking help from” [50]. Results suggest that some parents may be completely unable or unwilling to assimilate study information, with many parents reporting that they could not remember anything they had been told about the trial, and even that they were “not listening to anything” whilst receiving study information [50]. Only 18 % of parents interviewed fully recalled the nature of the research they had agreed to their child participating in [50].

Vulnerability refers to an inability to protect oneself, due to intrinsic and situational factors that threaten voluntary choice [25, 69]. In this context, the critically ill or injured child’s parents and family should also be seen as being vulnerable [18, 53]. Parents are required to make decisions for their child at a time of intense emotional stress, in a foreign environment [70] with the potential for perceived coercion (for example if the parent fears his/her child’s quality of care is contingent on participation in a trial), undue inducement and exploitation.

In many cases, therefore, it may be impossible and/or inappropriate to obtain, or attempt to obtain complete voluntary, informed consent for research participation in the context of a critically ill or injured child, owing to situational incapacity of the parent at a time of intense emotional stress. This situational incapacity could be a potential justification for considering alternative consent models [6].

Where possible, the research consent process should start before admission [11] when the parent is emotionally stable, but this is only feasible for elective admissions, for example for children admitted to intensive care units following surgical correction of congenital heart defects [71]. This approach is similar to that used in neonatal research where parents may be informed antenatally about potential recruitment of their newborn in emergency research, and are provided with the option of opting out (declining) at a time of relative emotional stability [72]. Elective admissions constitute the minority of South African PICU admissions [10]; therefore a pre-admission consent model would only be appropriate or possible in specific research contexts.

#### “First do no harm”

Health care providers have an obligation to relieve and prevent suffering of patients and their relatives. The added burden for parents of being approached to provide consent for research as well as consent for clinical care at a time of intense stress, has been discussed previously [43, 50, 54, 68, 73]. Ethically, respect for persons (including parental autonomy) must be respected and the safety of the child-patient must be ensured, which includes safeguarding against the potential negative effects of decision-making in a highly stressful medical environment [43].

The requirement for consent may also add strain on patient (parent)/clinical healthcare provider relationships. For example, nurses responsible for patient clinical care may not be able or allowed to provide directive advice about study enrolment or procedures, particularly in a high-acuity emergency situation [50].

A study of parents’ perspectives on consent approaches for minimal risk research in hospitalised children reported an increased level of anxiety as a negative effect of a written consent approach; and also reported that having to sign a consent form resulted in the research being perceived as being more risky than when verbal consent was requested [74].

In this context, the burden (in terms of increased anxiety) to the parent of being asked to consent to their child’s enrolment in low risk research may be seen as introducing harm, without adding to the benefits of the research.

#### Sociocultural issues

In some non-Western cultures, including traditional African cultures, it may not be considered appropriate to obtain individual informed consent [75–77]. Gender and generational hierarchies inform family decision-making in many South African communities. In some cases women may not have societal authority to make independent decisions for their children, instead requiring assistance and agreement from other family members, including the husband (father) and the child’s grandparents [78]. If the parent so wishes, it might therefore be more respectful to inform the extended family and/or community about the research, with the ultimate decision about participation still made by the legal proxy as required by South African law [77]. This again requires time, which might not be available given the child’s severity and acuity of illness.

Cultural norms and behaviours may also threaten the validity of consent. For example, people from some cultural backgrounds in South Africa might respond with an affirmative to any question requiring choice. Although this might indicate understanding, in some cases it merely indicates respect for the person in authority – acknowledging a lack of understanding might suggest that the researcher had not provided adequate explanation, which could be seen as being disrespectful [77].

The MalariaGEN study also reported that having a well-written consent form does not guarantee understanding, especially in populations with high levels of illiteracy and where indigenous languages do not include research-related terms. In such situations parents may rely on verbal explanations during the consent process [49].

#### Therapeutic misconception

Research, which aims primarily to generate generalisable knowledge for future patients, should be distinguishable from clinical care, which is focused on providing benefit to individual patients [3, 79]. Failure to understand the distinction between research and clinical interventions is termed “therapeutic misconception”, and could lead to questionable or invalid consent [53, 80].

Examples of therapeutic misconceptions include the belief that randomisation is a way of rationing access to scarce or expensive medical technologies; that the child participant would always directly benefit from the research interventions; and that participation ensures access to certain medications or therapies not otherwise available, despite potential randomisation to placebo arms [3, 11, 17, 50, 53, 80–85]. Parents of children with life-threatening illness or injury may be more likely to consent to participation in clinical trials in the hope of finding a “miracle cure” [86].

Parents of critically ill or injured children usually have to fill in a number of consent forms for clinical procedures. In South Africa and other low and middle-income countries, these include consent for surgery, blood administration, HIV testing, use of restricted medications, and vascular access procedures. In this context it is challenging to ensure that parents completely understand whether consent is being given for normal therapeutic activities or research. A study of consent options for paediatric critical care research reported that 16 % of parents were unlikely to properly read a research-related hand-out because they felt overwhelmed by the sheer volume of papers they had received [87].

There may also be confusion and the potential for undue influence when clinician-researchers fulfill a dual role [3, 83, 88]. It has therefore been suggested that an independent third party should explain the research and obtain informed consent [3], although this might also have drawbacks in having an unknown person approach the parent at an emotionally vulnerable time. Informed consent forms should avoid using terms that might aggravate therapeutic misconception such as “doctor”, “treatment”, and “medication” [79].

In certain cases the term “misconception” is a misnomer, as children enrolled in some clinical trials may actually receive better care than those not enrolled. A number of clinical trials in Africa have reported that critically ill children enrolled in trials were more frequently assessed, had closer monitoring, and parents did not have to pay for some aspects of their care, which would have been required if the child were not enrolled [50, 89]. Indeed, in some poorly resourced settings, clinical trials provide the only access to quality health care services for the local population [90]. Patient outcomes for children enrolled in both the therapeutic and control groups of clinical trials have been noted to be substantially better than the expected outcome for children admitted with the same medical status outside the trial [89, 91].

### Informed consent – principles and options

*“…it is dangerous to let one moral principle – informed consent – become absolute” -* M. Bland, 1997 [92].

Respect for autonomy is often considered the most important ethical principle in medical research ethics [93]. Informed consent aims to ensure respect for participant autonomy by promoting choice based on individual values, to avoid participants being used merely as a means to an end. This is particularly important in a situation where researchers’ interests may differ from the aims of clinical care [45, 79]. Informed consent is not just obtaining a signature on a form, it is a process of information exchange between the investigator and potential participant, with the latter receiving enough information to be able to make voluntary, fully informed decisions about whether or not to participate [3, 79, 94, 95]. Specific components of the informed consent process have been outlined previously [94].

Most critically ill children cannot be considered autonomous, as they are incapable of understanding, communicating and/or making meaningful choices or decisions [93]. The principle of respect for participant autonomy may, therefore, not be directly applicable to these children [93], but the overriding principle of respect for persons holds [21].

It has been suggested that in order to properly protect children in paediatric critical care research, the primary emphasis on respect for parental autonomy (and therefore informed consent) should be replaced by a focus on the other ethical principles of beneficence, non-maleficence, and justice, whilst maintaining respect for the child’s developing or future autonomy [5, 26, 43, 93]. Non-maleficence includes limiting or preventing physical and psychological threats, for example by minimising research risks [5, 43]. The South African Constitution states, ‘a child’s best interests are paramount in every matter concerning a child’ [96]. Acting in the child’s best interests involves the combined principles of beneficence and non-maleficence [93], and these principles are supported by local RECs’ essential role in filtering out research protocols with an unacceptable risk: benefit profile so that parents are only asked to consider participation in appropriately vetted, safe studies (the Nuffield “fair offer” concept) [97, 98].

RECs should therefore initially act *in loco parentis* by only approving those studies that the most conscientious parent would agree to [97, 98], as well assuming a *parens patriae* role in protecting those who cannot protect themselves [31]. In this way, RECs can ensure that important paediatric research proceeds appropriately and with sufficient protection of vulnerable participants, regardless of whether parental consent can be consistently or adequately obtained. It has, however, also been argued that research governance may be overly restrictive and paternalistic. In the case of severe paediatric disease, parents may allow their children to participate in trials despite high levels of risk, and may even see this as a right [99]. In order to prevent RECs from acting as the sole arbiter of what is in the participants’ best interests, community consultation or collaboration, including parents of previously critically ill children, medical professionals and REC members, for example, is recommended to ascertain what could be considered acceptable levels of risk.

Chapter 9 (section 71) of the South African National Health Act 61 of 2003 presents consent to participation in research as a statutory imperative, and does not allow for deferred or waived consent under any circumstances [34, 37]. South African law does not, therefore, adequately address the complex ethical and procedural issues relating to paediatric critical care research, and should be challenged as such.

Blind adherence to the legislative and ethical doctrine of informed consent, without true consideration for the underlying ethical principles of beneficence *and* respect for individual autonomy, may promote deceitful or inadequate consent mechanisms, which are not in research participants’ best interests [45]. Attempting to obtain informed consent where it is largely impossible is demoralising and threatens the integrity of the researcher, REC and the institution [45]. Considering options other than prospective, informed consent in carefully selected cases along with appropriate local REC oversight and research risk reduction, may be ethically preferable [83].

If alternatives to the prospective informed consent model are never considered, many important studies could not feasibly be conducted, and unproven therapies would continue to be used in the care of critically ill and injured children. By considering alternatives and challenging restrictive legislation, South African RECs would be fulfilling their role as advocates for vulnerable child-participants and their parents [53].

### Options for consent

#### Prospective informed consent

It is generally agreed that all studies of novel investigational drugs, devices or biological agents require comprehensive, voluntary, prospective informed consent [13]. Randomised controlled trials often compare the best standard treatment with a new treatment, which it is hoped might be more effective. The new treatment, however, might also be less or equally effective, and it may be associated with unacceptable adverse events. Parents should be informed of all possible risks and benefits, even if doing so raises anxiety and adds stress [53].

Children require permission from a parent or legal guardian to participate in clinical trials [22, 35, 36]. This depends on the concept that parents are best placed to make medical decisions for their children, which is not always the case [12, 23, 93]. In order to make decisions for their children, parents or legal guardians should ideally display commitment to the child’s best interests; adequate knowledge and information; emotional stability; and the ability to make reasoned judgements [100]. Only a parent with decisional capacity can give truly informed consent [22].

Even if the parent has sufficient capacity, parental choice cannot reflect the views of a young child or infant - at best it reflects parental discretion and family values [53]. Therefore, assent should be sought wherever possible in awake, aware children of sufficient age and developmental level to understand basic research concepts and implications [12, 21, 42, 101, 102]. The assent process is a way of appropriately engaging with the child about his/her participation in research, thereby showing respect for their developing autonomy and sense of self-worth [103]. Realistically, obtaining assent is seldom possible in the critical care environment but whenever possible, assent should be taken in a developmentally appropriate manner [22] and may require alternative communication methods in ventilated children, for example. The assent process may give the child a sense of control over their own bodies and a voice in a situation where they often have none [12]. Dissent should always be respected for research participation, regardless of parental consent, for all children with sufficient developmental levels of understanding and decision-making ability [5, 22, 25].

Where prospective informed consent is required and appropriate, this should involve both a verbal and written or otherwise recorded communication process, ideally in the parent or legal guardian’s home language [82]. Careful reading of shorter, simplified forms, written at a suitable reading level, has been shown to enhance understanding [79, 87, 104–106].

Assessing parental understanding is fundamental to the informed consent process, and can be done using open-ended questions after comprehensively describing the research, such as, “how will being part of this study help your child?” Leading and closed-ended or yes/no questions, such as, “do you understand what this research is about?” should be avoided [25, 107].

It is recommended that where prospective informed consent is required by the local REC, the quality of consent (capacity, voluntariness, understanding) be assessed or confirmed by independent consent monitors, wherever feasible [25, 44, 108].

*Waiver of individual consent, deferred consent, and “blanket assent”*

“*When a child arrives there, let him/her be treated first and then later let [the parents] be asked the questions*”. Quote from parent of child participant in the Fluid Expansion as Supportive Therapy (FEAST) Trial [50].

Research in the context of critically ill or injured children may be considered ethically justifiable without prospective voluntary informed consent if it has the potential to benefit the population by producing sound evidence or a better understanding of best treatment (i.e. is important, relevant and scientifically sound), and/or where the intervention has only minimally increased risk in the context of the child’s underlying condition [6, 27].

### Waiver of prospective informed consent

The United States Common Rule states that an ethics review board may waive the need for informed consent if the research involves no more than minimal risk to participants; the waiver would not adversely affect the rights and welfare of the participants; the research could not practically be carried out without the waiver; and, whenever appropriate, participants would be provided with pertinent information after participation [20]. Other groups have provided similar guidelines [109].

A systematic review of 11 paediatric studies focussing on clinical research without prospective consent suggested general support for waiver of consent in acute care research [13]. However, providing no study information to the parent precludes the option of opting out, and thus ignores the many reasons why a parent would not wish their child to participate in a clinical study [13]. Waiving the need for informed consent may diminish parental autonomy and undermine public trust in the healthcare service and clinical research [50], however this model might be appropriate for very low risk studies such as folder reviews, observational studies of standard practice with non-invasive outcome measurement, and practice improvement initiatives [50, 110].

A paediatric emergency resuscitation study, involving a trial of brain cooling after in-hospital arrest, reported that the majority of parents supported the research occurring with a waiver of prospective informed consent, as long as they were aware of the research study in progress and had the opportunity to “opt out” [87, 111]. Parents also endorsed a community consultation process for paediatric critical care research [87]. Families of children sharing a particular disease, including the experience of critical illness or injury, form a meaningful community [88, 112]. Therefore appropriate community consultation could include dissemination and display of research information, such as brochures and posters, within existing support and information-sharing groups of parents of critically ill or injured children. These materials should summarise the purpose, risks and benefits of the study/studies being conducted in the unit; state that no formal individual consent would be sought; present ways to opt out of the research; and direct parents to the contact person should they have questions or to give feedback [112]. Such a “blanket assent” approach has been shown to be effective and acceptable to parents in a prospective drug trial for paediatric cardiopulmonary arrest, with 81 % of parents being aware of an ongoing trial and only 9 % indicating that they would not want to participate [112].

Transparency and communication are essential components in creating trust and protecting autonomy. All reasonable efforts should be made to maximise communication with research participants and their families in as timely a fashion as possible [87], while being respectful of not over-burdening parents with unnecessary or inappropriate information. In this context, “saturating” the hospital environment with information about on-going research studies may reduce anxiety and improve understanding and trust [87]. High illiteracy levels in the South African context may limit the efficacy of this approach and alternatives using verbal communication, such as focus support and information sharing groups, should be considered in addition to written material.

### Critical care audits and practice improvement initiatives

Critical care audits and practice improvement initiatives are gaining popularity as an evidence-based approach to improving the care and outcomes of critically ill and injured children. An example is the implementation of practice “bundles” and continuing audit to reduce the incidence of hospital-acquired infections in PICU, as undertaken by the Institute for Healthcare Improvement (http://www.ihi.org/Engage/Initiatives/Completed/5MillionLivesCampaign/Pages/default.aspx).

It is generally accepted that these initiatives should form part of clinical practice; but when the results are sufficiently important to warrant dissemination, ethical regulations, particularly the requirement for prior review and informed consent, may appear unnecessarily restrictive. The concept of “learning health systems” has therefore been proposed [113], in which high-quality patient care is combined with routine data collection, without systematically modifying therapy for the purpose of developing generalisable knowledge [113]. This blurs the distinction between practice and research, which has been upheld since the *Belmont Report* [21]. “Learning health systems” might be particularly important in resource-limited countries such as South Africa, where identification of which aspects of critical care management impact on patient outcomes can inform appropriate utilisation of scarce hospital resources. Unnecessary regulatory burdens could impede this process, with potential negative consequences to patients.

Obtaining informed consent from every child admitted to PICU is not practical, and even if sought, missing data from those unable to provide consent for involvement in practice improvement initiatives could substantially skew the data. Waiving consent is justifiable in this context as no experimental intervention is assigned, so everything studied could be seen to fall under standard medical care, with no added risk [113]. It is generally accepted however, that even if formal consent is not sought, parents should be informed about on-going projects [113], for example by focus group discussions, posters, and dissemination of brochures, as mentioned previously [114].

### Deferred consent model

*“In my case it was after my child felt better the following day before they came and had the discussions with me. If the girl [project staff] had come on the day I brought the child I could not have listened to her”.* Mother of participant in the MalariaGEN trial [49].

If one accepts that informed consent is often not truly possible in the paediatric critical care context, obtaining approval in principle (assent) would allow informed consent, when needed, to be deferred and the process continued until parents are available and/or competence is achieved [6]. This is the model for deferred, or continuing consent: a child is enrolled in a study, preferably with limited explanation and verbal assent; but informed consent for continued enrolment in the study is deferred until the child has stabilised and/or the parent or guardian is able to assimilate study information sufficiently to make an informed decision [6, 50]. Initial assent refers to an affirmative agreement indicating some level of understanding and decision making ability, and essentially provides a quick opt-out option before study interventions are performed [50]. There is currently insufficient empirical evidence on whether it is preferable to provide limited study information in an initial assent process, or rather to completely defer all information and obtain consent to use data already collected only once the child’s condition has stabilised and/or the parent has regained capacity.

Deferred consent, as used in the “FEAST” and “MalariaGEN” trials, might be useful in emergency situations where urgent action is required and prospective informed consent is not reasonably practical [6, 49, 50, 68]. Item 30 of the Declaration of Helsinki (2013) approves the concept of deferred consent if the legally authorised representative is not available and the research cannot be delayed. “The study may [then] proceed without informed consent…[and] consent to remain in the research must be obtained as soon as possible” [9]. The concept of deferred consent in critical care research is generally supported by parents and clinical and research staff [49, 50, 115].

Further ethical dilemmas arise when an enrolled child dies before deferred informed consent can be obtained: Should bereaved parents be approached for consent after the child’s death and should their collected data be included in the analysis [11]? Excluding data on children who die before consent can be obtained could introduce systemic bias; reduce statistical power; decrease external validity; and skew randomisation, thus threatening scientific validity of the study [11, 95, 116, 117]. The intention to treat principle also requires that all randomised participants be included in the primary analysis [95]. A randomised controlled trial of critically ill adults found that when participants without deferred consent were excluded from analysis, there was no treatment effect (p = 0.35), but this became significant (p = 0.006) when all participants were included [116]. Thus, not including those without complete deferred consent could cause harm by decreasing study validity [116, 117]. This could be considered unethical and unjust to those participants who were enrolled in the trial with consent [116].

Confronting recently bereaved parents with requests for deferred consent could unjustifiably add harm by increasing parental distress with no possible benefit to the child participant [95, 118]. Furthermore, the high emotional stress levels of a recently bereaved parent might again render them situationally incapacitated, which would make it inappropriate to approach them for informed consent at that time [95].

The “FEAST” trial did not seek consent for included children who died in order to reduce parental self-blame [50, 68]. Diverse staff interviewed generally felt this approach was humane and appreciated, but the need for further discussion and reflection, including in-depth consultation of community members, was noted [50]. Another study in a different context reported that two thirds of parents of deceased children *would* want to be informed about the trial they had been enrolled in [115]. There may be a large distinction between being informed of a study and providing comprehensive informed consent after the event. On consideration of the risk: benefit balance, it is suggested that clinical data collected on children who die before deferred consent has been obtained should be included, as long as confidentiality and privacy requirements are met [95, 117, 118].

Research ethics committees should assess trial protocols individually to determine the appropriateness and methods of using deferred consent. This process should ideally also be informed by community consultation with parents of children who have received or are receiving critical care; by asking them hypothetically whether they found the proposed study and consent method acceptable [87, 88, 112]. It is further recommended that empirical research involving bereaved parents be conducted in South Africa in order to objectively inform consent models in this context.

### Comparative effectiveness research

In the absence of good evidence about which competing, but generally accepted therapy or equipment is best, many clinicians choose care practices on the basis of advertising, local custom and personal preference [119]. This can lead to harm by administering untested and unproven interventions [113]. The premise behind comparative effectiveness research, often using interventional randomised controlled trial designs, is that being assigned to either treatment group exposes no additional risk to the patient, assuming both fall within the range of standard clinical practice. This is different from the inherent risk of being randomised to the experimental arm of a trial assessing a novel intervention [119]. In comparative effectiveness research, any incremental risks would arise from nontherapeutic trial procedures, which are usually limited to recording clinical data and analysing biological samples. Therefore, these studies generally only pose minimal risk [7] and may even be considered as such when testing two drugs (assuming both drugs could have been routinely given as part of standard care). Cluster-randomised trials are often used in comparative efficacy studies, with interventions delivered across an entire unit or institution. This makes obtaining individual informed consent impossible in some cases, although permission to use the data can generally be obtained, along with information dissemination using methods outlined above [27].

## Summary

The primary ethical responsibility in paediatric critical care research is to protect this vulnerable population from exploitation and harm. However, there may be considerable harm arising from the sometimes-restrictive ethical requirements imposed by RECs according to current South African laws and norms. These requirements, specifically those demanding informed consent in all situations, although meant to ensure protection, may be impractical, inappropriate, or even impossible to correctly obtain.

It is our contention that parents and legal guardians of critically ill or injured children should only be approached for consent to participate in clinical research when it is ethically appropriate to do so [95]. South African RECs should, therefore, be permitted to consider alternatives to prospective informed consent where it is in the best interests of participants and their parents, and/or without which important research might not be able to be undertaken, whilst being cognisant of the risk levels of the proposed research. There also needs to be an appreciation that even some randomised controlled trials might afford minimal incremental risk to participants. Alternative consent options include waived or deferred consent when the research is of emergency care and/or the parent is incapable of providing fully informed consent owing to high levels of distress [50, 68].

The challenges endemic to research in the paediatric critical care population warrant the development and amendment of specific South African regulations and guidelines to clearly define the conditions under which research can legally, ethically and realistically be conducted without prospective informed consent [45, 120]. This process should be informed by local empirical research and community consultation. Having clear and consistent guidelines would facilitate local REC deliberation and consensus; enhance research integrity; and show a clear way forward for paediatric critical care research in South Africa.

## Abbreviations

REC: Research ethics committee
PICU: Paediatric intensive care unit
GCP: Good clinical practice
HIV: Human immunodeficiency virus
